# In Vitro and In Vivo Evaluation of Silver Nanoparticles Phytosynthesized Using *Raphanus sativus* L. Waste Extracts

**DOI:** 10.3390/ma14081845

**Published:** 2021-04-08

**Authors:** Camelia Ungureanu, Irina Fierascu, Radu Claudiu Fierascu, Teodora Costea, Sorin Marius Avramescu, Mirela Florina Călinescu, Raluca Somoghi, Cristian Pirvu

**Affiliations:** 1 Department of General Chemistry, University “Politehnica” of Bucharest, 011061 Bucharest, Romania; cristian.pirvu@upb.ro; 2 National Institute for Research & Development in Chemistry and Petrochemistry—ICECHIM, 060021 Bucharest, Romania; irina.fierascu@icechim.ro (I.F.); raluca.somoghi@icechim.ro (R.S.); 3 Faculty of Horticulture, University of Agronomic Sciences and Veterinary Medicine of Bucharest, 011464 Bucharest, Romania; 4 Department of Science and Engineering of Oxide Materials and Nanomaterials, University “Politehnica” of Bucharest, 011061 Bucharest, Romania; 5 Phytotherapy Department, Faculty of Pharmacy, Pharmacognosy, Phytochemistry, University of Medicine and Pharmacy “Carol Davila”, 020956 Bucharest, Romania; teodora.costea@umfcd.ro; 6 Research Center for Environmental Protection and Waste Management, University of Bucharest, 050107 Bucharest, Romania; sorin_avramescu@yahoo.com; 7 Research Institute for Fruit Growing Pitesti—Mărăcineni, 110006 Pitesti-Mărăcineni, Romania; elacalinescu@yahoo.com

**Keywords:** phytosynthesized AgNPs, radish extracts, antifungal activity, antimicrobial activity

## Abstract

The aim of the current paper is the development of phytosynthesized silver nanoparticles mediated by *Raphanus sativus* L. extracts obtained through two extraction methods (temperature and microwave) and to test their potential application for controlling apple crops pathogens. The phytosynthesized materials were characterized by X-ray diffraction, scanning electron microscopy, and transmission electron microscopy. All the materials were evaluated in terms of antioxidant and in vitro antimicrobial activity (against bacteria, molds, and yeast: *Escherichia coli* ATCC 8738, *Staphylococcus aureus* ATTC 25923, *Pseudomonas aeruginosa* ATCC 9027, *Salmonella typhimurium* ATCC 14028, *Candida albicans* ATCC 10231, *Venturia inaequalis*, *Podosphaera leucotricha*, *Fusarium oxysporum* ATCC 48112, *Penicillium hirsutum* ATCC 52323, and *Aspergillus niger* ATCC 15475). Considering the results obtained in the in vitro assays, formulations based on nanoparticles phytosynthesized using *Raphanus sativus* L. waste extracts (RS1N) were evaluated as potential antifungal agents for horticultural crops protection, against *Venturia inaequalis* and *Podosphaera leucotricha* through in vivo assays. For the DPPH assay, the inhibition (%) varied between 37.06% (for RS1N at 0.8 mg/mL concentration) and 83.72% (for RS1N at 7.2 mg/mL concentration) compared to 19.97% (for RS2N at 0.8 mg/mL) and only 28.91% (for RS2N at 7.2 mg/mL). Similar results were obtained for RS3N (85.42% inhibition at 7.2 mg/mL) compared with RS4N (21.76% inhibition at 7.2 mg/mL). Regarding the ABTS assay, the highest scavenger activity values were recorded for samples RS1N (91.43% at 1.6 mg/mL) and RS3N (96.62% at 1.6 mg/mL).

## 1. Introduction

Nowadays, nanotechnology and nanomaterials are applied in different areas, as electronics, photonics, or energy industries, textiles, environmental protection, food and agriculture, biomedicine, or health care [1]. Recent progresses in the nanotechnology field reveal the uses of nanomaterials for developing new formulations, based on natural active compounds obtained from different plants, which are an inexhaustible source of bioactive molecules [2]. Expensive methods such as plasma or flame spraying, laser pyrolysis, atomic or molecular condensation, sol-gel, chemical vapor deposition, precipitation, microemulsion, and sonochemistry, as well as environmentally hazardous reagents (sodium borohydride, Triton X, sodium formaldehyde sulfoxylate etc.) can be replaced for the obtaining of nanomaterials with controlled morphology and properties, with ecofriendly methods based on the use of active compounds from plants and plants waste [3]. Most of the obtained materials based on natural extracts from different plant species proved to be a valuable tool in various bio applications [4,5].

Silver represents one of the most well-known materials with antimicrobial properties, ever since ancient times. In the same, the modern problems (such as development of bacterial resistance to classical antimicrobial agents) led to the reconsideration of silver as an alternative therapeutic option [6]. In its “nano” form, silver was proven to be an important candidate for application in biomedicine, in applications such as antimicrobial, anticancer, or anti-diabetic therapy, wound repair and bone healing, or as biosensors [7]. Their application exceeds this area, silver nanoparticles (AgNPs) being used in different other areas, included, but not limited to catalysis, microelectronics, wastewater treatment, or renewable energy [8,9].

The emergence of green chemistry has drawn researchers’ attention to develop alternative ways to produce different types of materials, such as metallic nanoparticles due to the involvement of environmentally friendly reducing agents and non-toxic substances for the nanoparticles’ stability [10,11,12,13]. Additionally, the capitalization of horticultural wastes has important environmental implications, decreasing the amount of wastes, and, at the same time, obtaining useful bioactive compounds. Most of the literature concerning the re-utilization of horticultural plants wastes is related to their use as compost material [14]. These large amounts of waste can produce pollution and economic loss and causes landfilling to be no longer sustainable [15]. Agro-industrial side streams are an exhaustless source used for obtaining nanomaterials with further applications: for example, cocoa pod husk can be used for obtaining silver nanoparticles with larvicidal activities [16], mango peel for obtaining gold nanoparticles with anti-cancer properties [17], grape seeds or sweet osmanthus leaves for antimicrobial silver nanoparticles [18,19], Chinese holly leaves applied in the phytosynthesis of silver nanoparticles for development of antimicrobial textiles [20], rambutan peels for the synthesis of ZnO nanocrystals with antibacterial applications [21], the rinds of *Garcinia mangostana* L. for obtaining silver, gold, and platinum nanoparticles [22], etc., the biosynthesis of NPs using waste materials being an emerging and upcoming area of research [23].

The mechanism of nanoparticles phytosynthesis is well-known, involving the use of phytoconstituents both as reducing and capping agents [23,24]. Cruciferous vegetables represent a good source of active compounds as a base for further applications. From cruciferous vegetables, *Raphanus sativus* L. (radish) represents a very important vegetal crop all over the world, widely consumed as a condiment or a vegetable in human diets [25]. The aerial part of radish (leaves) has long been grown as a feed crop, but it also has various medicinal actions, harvesting their antimicrobial and antiviral [26], or antioxidant potential [27]. The main components of *Raphanus sativus* L. leaves are known as phenolic compounds [28], with a relatively high content of flavonoids as quercetin and luteolin. Their application for the phytosynthesis of nanoparticles represents the focus of significant fewer works, generally based on the synthesis of nanoparticles such as ZnO [29,30].

To sustain the food needs of the ever-growing population and since the crop yield and quality are adversely affected by plant diseases (caused by different types of microorganism, including bacteria, viruses, and especially fungi), which have lately become more virulent and more resistant to synthetic fungicides [31], nanomaterials developed by eco-friendly routes represent a sustainable alternative to control pathogens and overcome the shortcomings of conventional pesticides. Several studies based on novel nanomaterials have been developed for controlling apple crop diseases [32,33,34,35], but up to our knowledge, in scientific literature, there are no studies regarding phytosynthesised silver nanoparticles using different extracts obtained from the leaves of *Raphanus sativus* L., having antioxidant and antimicrobial properties, with practical applications in treating diseases that affect apple crops. The economic importance of the apple cultures (*Malus pumila* Mill.), one of the most economically relevant fruit crops in the world mainly due to a wide range of dietary products deriving from it [36], and the increased consumer’s demands for safe and secure products [37], led to the development of new formulations able to treat the diseases that affect these crops.

By using the proposed method (involving plant wastes) for the development of valuable nanostructures, residues from primary and secondary processing are minimized and reutilized, contributing to the sustainability needs of new materials. The aim of the present paper is to develop new recipes based on phytosynthesized silver nanoparticles mediated by *Raphanus sativus* L. extracts obtained through two extraction methods (temperature and microwave) and to test their potential application for controlling apple crops pathogens. The phytosynthesized materials were characterized by X-ray diffraction, scanning electron microscopy, and transmission electron microscopy. All materials were evaluated in terms of antioxidant and in vitro antimicrobial activity (against bacteria, molds, and yeast: *Escherichia coli* ATCC 8738, *Staphylococcus aureus* ATCC 25923, *Pseudomonas aeruginosa* ATCC 9027, *Salmonella typhimurium* ATCC 14028, *Candida albicans* ATCC 10231, *Venturia inaequalis*, *Podosphaera leucotricha*, *Fusarium oxysporum* ATCC 48112, *Penicillium hirsutum* ATCC 52323, and *Aspergillus niger* ATCC 15475). In addition, selected materials were evaluated as potential antifungal agents for horticultural crops protection, against *Venturia inaequalis* and *Podosphaera leucotricha* through in vivo assays.

## 2. Materials and Methods

### 2.1. Preparation of Plant Extracts

Leaves of *Raphanus sativus* L. were harvested from Dărăști-Vlașca area, Giurgiu County (44°17′30″ N 26°0′34″ E), grown from certified seeds. Multiple specimens were collected during summer (June–July) and a representative voucher specimen was deposited in Argeș County Museum Herbarium, Pitești for future reference (voucher no. 11327/10.07.2018). Part of the plant material was preserved (−18 °C) in the General Chemistry Department, Faculty of Applied Chemistry and Materials Science, Bucharest. The fresh leaves were washed thoroughly in tap water, rinsed well in distilled water, pat dried with paper towel until all water molecules present were completely evaporated, and chopped at room temperature. Subsequently, the leaves were shade-dried to constant mass [38].

The parameters of the extraction procedures were classical extraction: extraction time—3 h., mechanical stirring, extraction temperature—70 °C (hydroalcoholic mixture), 67 °C (ethanol); microwave-assisted extraction: extraction time—10 min. at 140 °C, maximum power 1000 W.

In order to obtain the hydroalcoholic extract by classical thermal extraction (encoded as sample RS1), 50 g of dried upper aerial part of the plant were mixed in 600 mL 1:1 water-ethanol, at 70 °C for 3 h, under mechanical stirring. The ethanol used was analytic grade (Merck KGaA, Darmstadt, Germany), while the bidistilled water used in all the experiments was obtained in the laboratory (using a GFL 2102 water still). Ethanolic extract (RS2) was prepared using ethanol as extraction solvent, at 67 °C, for 3 h, under mechanical stirring.

The microwave-assisted extraction was performed in an Ethos Easy Microwave Solvent Extraction Lab station (Milestone, Sorisole, Italy), with the following parameters: step 1: 20–140 °C for 5 min; step 2: 140 °C for 10 min.; step 3: cooling to room temperature; maximum power—1000 W; extraction solvents: hydroalcoholic solution (RS3) and ethanol (RS4), respectively; ratio vegetal material to solvent was kept at 1:12 *w*:*v*. The obtained extracts were filtered using Whatman Filter Paper Grade 3.

### 2.2. Evaluation of Chemical Composition of Radish Leaf Extracts

To evaluate the leaf extracts composition, the solutions were characterized using specific procedures for the determination of total phenolics content [39] and high-performance liquid chromatography (HPLC).

#### 2.2.1. Evaluation of Total Phenolic Content of the Extracts

The quantification of the total phenolic content of the extracts was determined using Folin–Ciocalteu reagent assay, as previously exhaustively presented by our group [40,41], briefly discussed in the Supplementary Material (results obtained by applying Equation (S1), Supplementary Material). The calibration curve was constructed using gallic acid analytic standard (Sigma-Aldrich, Darmstadt, Germany). The experiments were carried out in triplicate.

#### 2.2.2. HPLC Analyses

The HPLC analyses were performed using a Varian system (Prostar 410 solvent delivery pumps, Prostar 335 DAD detector, Prostar 410 autosampler); data were analyzed with the Varian Workstation 6.3 software. The mobile phase consisted of solution A (water, acidified with 1% CH_3_COOH, respectively acetonitrile, acidified with 1% CH_3_COOH. The applied flow rate was 1 mL/min with an injection volume of 10 μL. Calibration curves were constructed for each of the analyzed compounds (R^2^  >  0.999), using commercially available standards: phenolic acids (chlorogenic acid, ferulic acid, rosmarinic acid) and flavonoids (rutin, quercetin and chalcone), all supplied by Merck KGaA, Darmstadt, Germany.

### 2.3. Phytosynthesis of Silver Nanoparticles

For the phytosynthesis of the silver nanoparticles (AgNPs), 100 mL of each extract (RS1–RS4) were mixed with 100 mL of 10 mM aqueous AgNO_3_ solution and incubated at room temperature for 30 min. The synthesis was completed upon the apparition of the specific ruby-red color [40]. The obtained nano-architectures were encoded RS1N–RS4N.

### 2.4. Nanoparticles Characterization

Nanoparticle’s characterization was performed using X-ray diffraction and morphological observations, in order to obtain information regarding the shape and the size of the phytosynthesized nanoparticles. The NPs were preliminary evaluated by UV-Vis spectrometry, using a Ultra 3660 UV-Vis equipment (RIGOL Technologies Inc., Beijing, China, optical resolution 0.5 nm), in the wavelength range 390–500 nm.

#### 2.4.1. X-ray Diffraction

X-ray diffraction (XRD) analyses were performed using a Rigaku SmartLab 9 kW diffractometer (Rigaku Corp., Tokyo, Japan, operated at 45 kV and 200 mA, Cu_Kα_ radiation—1.54059 Å), in 2θ/θ scanning mode, between 30 and 85° (2θ). The components were identified by comparison with ICDD entries.

#### 2.4.2. Morphological Observations

The evaluation of the morphological features of the nanoparticles was realized using scanning electron microscopy (SEM) and transmission electron microscopy (TEM). Examination of the surface and cross-sectional morphology of the samples surface were performed using Thermo Scientific™ FEI Quanta™ 650 FEG variable-pressure and environmental high-performance scanning electron microscope ((Hillsboro, OR, USA, SEM with Field Emission Gun). Transmission electron microscopy images were recorded using Tecnai G2 F20 TWIN Cryo-TEM (FEI Company, Hillsboro, OR, USA), at 300 kV acceleration voltage and a resolution of 1Å.

### 2.5. Antioxidant Activity

The antioxidant activity of nanoparticles and their corresponding extracts was determined by means of well-known methods—the scavenger activity towards DPPH (2,2-diphenyl-1-picryl-hydrazyl), ABTS^•^+ (2,2′-azinobis-(3-ethylbenzothiazoline-6-sulfonic acid) free radicals and the ferric reducing power, the assays being exhaustively presented in the Supplementary Material (results calculated using Equations (S2) and (S3), Supplementary Material).

#### 2.5.1. DPPH Assay

2,2-diphenyl-1-picryl-hydrazyl (DPPH) is a stable free radical, at ambient temperature, presenting strong absorbance at 517 nm; in the presence of an antioxidant, it is reduced, the solution becomes yellow to colorless and the absorbance decreases. The assay was carried on according to the method proposed by Ohnishi et al. [42], as previously described [43,44]. The extracts concentration that inhibited 50% of the DPPH free radical (EC_50_, mg/mL) was determined graphically from the linear regression curve plotted between percent (%) of inhibition and solutions concentration (mg/mL). All measurements were performed in triplicate.

#### 2.5.2. ABTS^•^+ Radical Cation Scavenging Assay

The assay was carried on according to the method presented by Re et al. [45], as previously described [46,47]. The extracts concentration that inhibited 50% of the ABTS^•^+ free radical (EC_50_, mg/mL) was determined graphically from the linear regression curve plotted between percent (%) of inhibition and solutions concentration (mg/mL). All measurements were performed in triplicate.

#### 2.5.3. Ferric Reducing Power Assay

The ferric reducing power was determined according to the Oyaizu method [46], as described by Popescu et al. [47]. The solutions concentration providing 0.5 of absorbance (EC_50_, mg/mL) was determined graphically from the linear regression curve plotted between absorbance and solutions concentrations (mg/mL). All measurements were performed in triplicate.

### 2.6. In Vitro Antimicrobial Activity

Gram-negative and Gram-positive bacteria were chosen considering their incidence on apple cultures, as well as their possible detrimental effect on human health [48]. In terms of food safety, species of the *Fusarium*, *Aspergillus*, and *Penicillium* genera are considered the most significant because they produce the great majority of known mycotoxins [49]; at the same time, *Venturia inaequalis* and *Podosphaera leucotricha* represent the most pathogenic microorganisms that affect apple crops. Many important information about the plant pathology aspects are described in the literature [50].

For the determination of in vitro antimicrobial activity of nanoparticles and extracts, was used the agar well diffusion assay for antibiotic susceptibility as detailed in previous studies [51,52]. The sensitivity of the microorganisms to the tested sample was established by the diameter of the inhibition zones according to Ponce et al. [53] as not sensitive (diameter under 8 mm), sensitive (diameter from 9 to 14 mm), very sensitive (diameter from 15 to 19 mm), and extremely sensitive (diameter above 20 mm). The biological strains used for this investigation were *E. coli*, *S. aureus*, *P. aeruginosa*, *S. typhimurium*, *C. albicans*, *F. oxysporum*, *P. hirsutum*, *A. niger* and *V*. *inaequalis*, and *P. leucotricha* isolated from fruit samples provided by the Research Institute for Fruit Growing Pitesti—Mărăcineni [50]. Each experiment was carried out in triplicate with three separate assay runs, using as negative control the solvent of the extracts (water: ethanol or ethanol) and as positive control commercial antimicrobials.

The bacterial strains were grown in Luria Bertani Agar (Miller, LBA), plates at 37 °C with medium composition: 10 g/L casein enzymic hydrolysate, 5 g/L yeast extract, 10 g/L sodium chloride, 15 g/L agar and the tested fungus was grown in Potato-Dextrose-Agar (PDA) (Ph. Eur.) with medium composition: potato peptone 4 g/L, glucose 20 g/L, and agar 15 g/L. Sterile LBA, Malt Extract Agar (MEA), and PDA plates were prepared by pouring the sterilized media in sterile Petri plates under aseptic conditions. The test organism (1 mL) was spread on agar plates. Wells were made at the size of 6 mm diameter, in the agar plates using the sterile borer. The wells with 50 µL of tested samples were placed on the inoculated plates. Similarly, each plate carried a blank well by adding solvent alone (water: ethanol and ethanol, respectively) to serve as a negative control. Positive control used was gentamicin sulfate (Sigma-Aldrich) (10 μg/mL) for bacterial strains, miconazole nitrate (Sigma-Aldrich) (30 μg/mL) for *F. oxysporum*, *P. hirsutum*, and *A. niger*; commercial antifungal products Captadin 80 WDG (Water Dispersible Granules) (1.5 g/L) (Arysta LifeScience) and Sulfomat 80 PU (3 g/L) (Mifalchim) were used as positive controls for *V. inaequalis* and *P. leucotricha*, respectively, at the concentrations recommended by the producers. The percent inhibition of the target microorganism was calculated according to the following Equation:

(1) $$I\;\left(\%\right)=\frac{I_{\mathrm{ZD}}-\mathrm{NC}}{I_{\mathrm{ZD}}}\times 100,$$

where I_ZD_—inhibition zone diameter, NC—negative control.

For the evaluation of minimum inhibitory concentration (MIC, the lowest concentration of the assayed antimicrobial agent that inhibits the growth of the tested microbial strains, expressed in μg/mL), for each of the tested microorganisms was used the broth dilution technique [54].

Briefly, serially diluted logarithmic concentrations of tested extracts ranging from undiluted samples to 1/16 dilutions were inoculated with standardized overnight cultures of the microorganisms and incubated at 37 °C [55]. The most recognized standards are provided by the The Clinical & Laboratory Standards Institute (CLSI) and the European Committee on Antimicrobial Susceptibility Testing (EUCAST) [56]. The MIC determination experiment was performed in triplicate. Standard deviation was calculated as the square root of variance using STDEV function in Excel 2010.

### 2.7. In Vivo Antifungal Activity

Cultivated apples were selected for the in vivo assays, as it represents one of the most important fruit crops at international level [57], and has a particular economic value at national level. To study the antifungal potential of the samples, young seedlings originated from “Idared” cvs. were inoculated in greenhouse with a suspension of 4.5 × 10^5^ conidia/mL of *V. inaequalis* or *P. leucotricha,* applied using a manual atomizer under controlled conditions (temperature 18–20 °C and humidity 80–100%). The conidial suspension was provided from the naturally infected leaves of apples of three varieties: “Idared”, “Starkrimson”, and “Golden Delicious” cvs. from the demonstrative apple plots of Research Institute for Fruit Growing Pitesti, Romania. The leaves were collected in 2018, from August to September, dried and kept in storage. The leaves were hydrated in distilled water and the inoculum of *V. inaequalis* or *P. leucotricha* was brought to 4.5 × 10^5^ conidia/mL. After primary infection, the treatments were applied, the experimental variants being tested in triplicate.

Scab symptoms were evaluated two weeks after inoculation according to the scale of Chevalier et al. [58]: 0 = no visible reaction; 1 = pit-point symptom; depression of 100–500 µm where the epidermal cells have collapsed; no subcuticular stroma; 2 = wide but shallow depressions; limited stroma formation; no sporulation; 3a = epidermal cells collapsed over large areas; close to the center the abundant mycelial stromata could produce conidiophores with a limited number of conidia; 3b = lesions are a network of mycelial strands; aborted conidiophores are mixed with normal conidiophores; sporulating chlorosis and sporulating necrosis occur; 4 = numerous conidiophores are often grouped in clusters and sporulate abundantly; the mycelial stroma forms a dense subcuticular network [58,59].

Seedlings from the same batch, 3–4 weeks old, were maintained under controlled conditions at a constant temperature of 20 °C and high humidity to assess the natural infections with powdery mildew *P. leucotricha.* They were evaluated according to the powdery mildew disease severity rating scale for leaves [58], where 0 = no infection; 1 = ≤1% infection; 2 = 2–5% infection; 3 = 6–20% infection, 4 = 21–40% infection; 5 = >40% infection; 6 = 100% infection [60,61]. Visual assessment of the powdery mildew leaf area infection was carried out with the aid of diseases rating scale (Supplementary Material, Table S1, Equations (S4) and (S5)) as proposed by Spencer [60].

### 2.8. Statistical Analysis

All experiments were carried out in triplicate, and the obtained data were analyzed for statistical significance using analysis of variance (one-way ANOVA) and Tukey test to determine significant differences among means. Significant differences were set at *p* ≤ 0.05. The results presented represent the mean ± standard error of mean (SE) of independent replicates.

## 3. Results and Discussion

### 3.1. Evaluation of Chemical Composition of Leaf Extracts

The general composition of the extracts was evaluated using the total phenolic content and the level of selected compounds, by HPLC (Table 1). The amount of total phenolic in the tested crude extracts of the leaves of *Raphanus sativus* L., measured by the Folin–Ciocalteau method, varied significantly from 4.49 to 12.42 mg GAE/g dry weight (see Table 1).

From the presented results, it can be concluded that the hydroalcoholic solvent represents a better alternative for obtaining biological active extracts, as proved by the higher content in total phenolics, as well as by the higher levels of marker compounds selected for the HPLC analysis. In the same time, although the HPLC analysis shows higher levels of phenolic acids in RS3, compared with RS1, the total phenolic content is significantly higher in RS1 (see Table 1); this could be explained by a higher diversity of phenolic acids extracted using the classical thermal extraction. These observations could also influence the antioxidant and the antimicrobial properties of the evaluated materials.

### 3.2. Phytosynthesized Nanoparticles Characterization

The preliminary evaluation of the NPs using UV-Vis spectrometry (Supplementary Material, Figure S1) supports the phytosynthesis success. Often, UV-Vis spectrometry can be applied to evaluate the dimensions of the NPs, by determining the position of the specific peak [4,24]. In the present case, the position of the plasmon resonance bands (439, 431, 460, respectively 457) would suggest the synthesis of NPs with dimensions between 40–50 nm (RS1N and RS2N, lower for RS2N), respectively between 60 and 70 nm (RS3N and RS4N, lower for RS4N). However, these values should not be considered definitive dimensions, due to the fact that, as previously presented by our group, the position of phytosynthesized AgNPs specific peaks can suffer a bathochromic shift from its “true” position, as a result of the influence of a series of factors, including the aggregation of NPs in larger clusters and the presence of different phytoconstituents [24]. As such, the NPs dimensions should be further confirmed using other techniques, such as XRD or electron microscopy.

**Figure 1:**
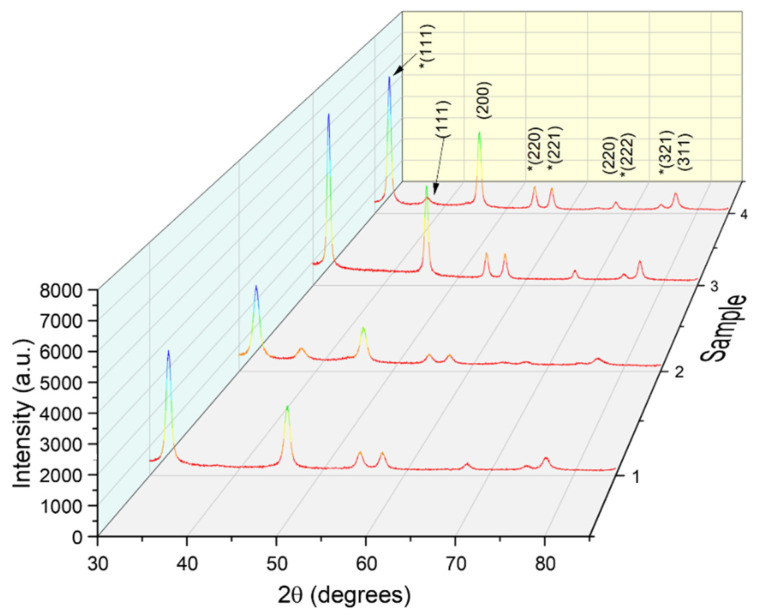
X-ray diffractograms of the obtained nanoparticles. Peaks marked with “*” are due to the presence of silver oxide.

The XRD analyses confirmed the phytosynthesis of the nanoparticles (diffractograms presented in Figure 1) and could also be applied for the determination of the average crystallite size (using the Debye–Scherrer equation, results presented in Table 2).

The diffraction peaks presented in Figure 1 confirm the synthesis of the AgNPs (ICDD PDF card no. 01-087-0720), alongside the silver oxide (ICDD PDF card no. 01-078-5867, marked on Figure 1 with asterisk), due to the oxidation of the NPs during sample treatment. From the results presented in Table 2, it can be noticed that the alcoholic extract leads to smaller dimensions nanoparticles, as did the classical temperature extraction. For the same solvent used, this could be an indicator of higher content in bioactive molecules (such as phenolic acids or flavonoids), as the phytosynthesis mechanism is closely related to the presence of these compounds [24].

**Figure 2:**
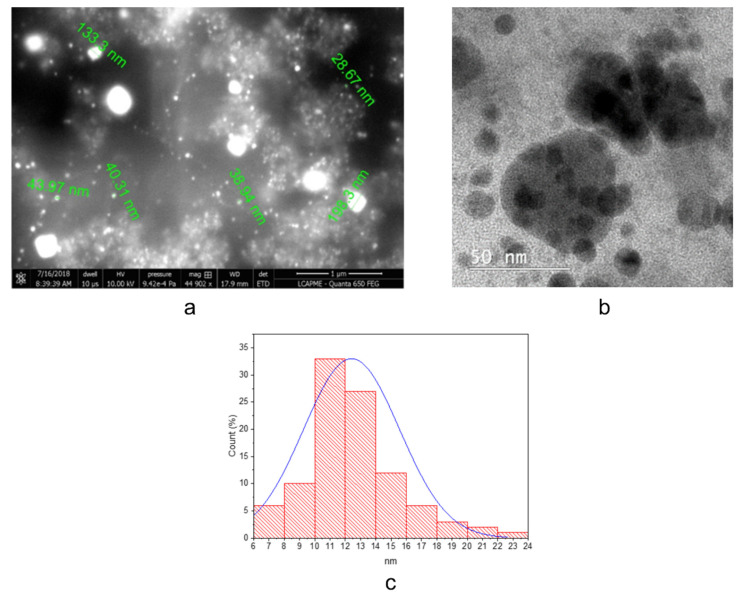
Morphological features of sample RS1N, as observed by SEM (**a**) and TEM (**b**) and size distribution of the particles, calculated from over 250 measurements from TEM images (**c**).

Morphological features of the obtained nanoparticles were evaluated using SEM and TEM techniques (representative images for sample RS1N are presented in Figure 2, while images of the other samples are presented in Supplementary Material, Figure S2).

Morphological observations suggest the phytosynthesis of spherical nanoparticles and, in some cases, agglomeration of particles with dimensions over 100 nm. The size distribution observed for all the samples are in good agreement with XRD data. The analysis of Figure 2c reveals that most nanoparticles observed for sample RS1N have dimensions between 10–14 nm, with the size distribution centered around 12 nm. The data presented in Table 2 shows for sample RS2N smaller dimensions, for most of the particles being in the range 9–12 nm, with the size distribution centered around 11 nm (see Supplementary Material, Figure S1c). The analysis of the microscopical images of samples RS3N and RS4N confirms the findings of XRD analysis, suggesting that using the microwave extracts (RS3 and RS4), the phytosynthesis procedure leads to higher dimension nanoparticles. A similar trend was observed regarding the solvent influence, as in the case of classical temperature extraction, the hydroalcoholic extraction leading to higher dimension nanoparticles, compared with the ethanolic extracts. For sample RS3N, the majority of the nanoparticles were in the range 10–20 nm (with the size distribution centered around 16 nm), while for sample RS4N, the majority of the measured nanoparticles were in the range 12–18 nm (with the size distribution centered around 15 nm). The observed characteristics of the nanoparticles were in concordance with the previous findings of our group [24,40], that the extraction procedure and solvent of choice leads to variation of the nanoparticles dimension (for the same vegetal material used), which, in turn, influences their biological properties [24].

Another major finding of the morphological observations is related to the shape of the nanoparticles: for all the extracts used, the majority of the nanoparticles were spherical in shape. This would support their potential antimicrobial applications, as several authors suggest the superior antimicrobial potential of spherical silver nanoparticles, compared with other morphologies (such as cubical, plate-shaped, or triangular) [62,63]. However, when speaking of phytosynthesized nanoparticles, the role of the extract’s composition is very important, the phytoconstituents contributing the final antimicrobial potential of the nanoparticles [24].

### 3.3. Antioxidant Screening

**Figure 3:**
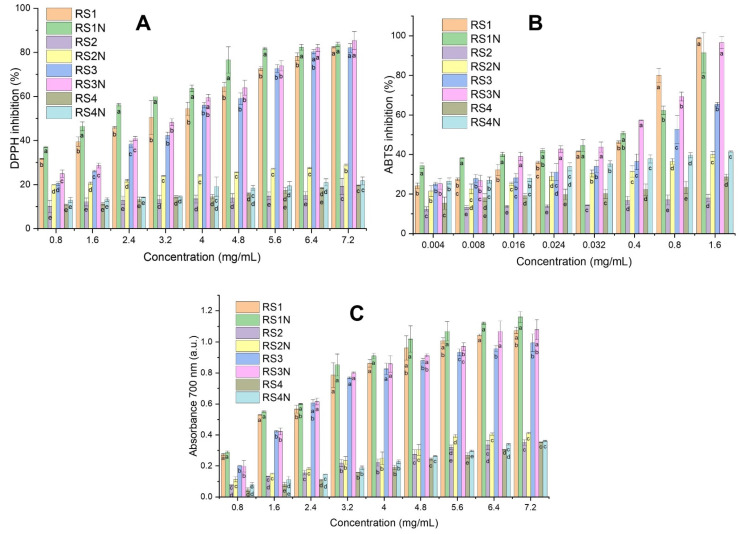
Antioxidant activity of analyzed extracts and silver nanoparticles: (**A**)—DPPH assay, (**B**)—ABTS assay, (**C**)—ferric reducing power. For each tested concentration, values without a common letter differ (*p* < 0.05) as analyzed by one-way ANOVA and the Tukey test.

For DPPH assay, the inhibition (%) recorded for the phytosynthesized nanoparticles varied between 12.94% (for RS4N at 0.8 mg/mL concentration) and 85.42% (for RS3N at 7.2 mg/mL concentration), respectively 83.72% (RS1N at 7.2 mg/mL concentration). However, comparing samples RS1N and RS3N, superior results were obtained for RS1N at all tested concentrations (except 7.2 mg/mL) (see Figure 3A). Regarding the ABTS assay, the scavenger activity varied between 21.5% (for RS2N at 0.004 mg/mL) and 96.62% (for RS3N at 1.6 mg/mL), respectively 91.43% (for RS1N at 1.6 mg/mL) (see Figure 3B). For ferric reducing power, the absorbance values varied between 0.11 (for RS2N at 0.8 mg/mL) and 1.16 (for RS1N at 7.2 mg/mL), respectively 1.08 (for RS3N at 7.2 mg/mL) (see Figure 3C). The DPPH scavenger activity and ferric reducing power were similar for RS1N/RS3N and RS2N/RS4N, respectively.

The analyzed nanoparticles and their corresponding extracts showed concentration-dependent antioxidant activity. Independent of the method used, the nanoparticles phytosynthesized using RS1 and RS3 extracts have a better antioxidant capacity compared to the ones obtained using RS2 and RS4 extracts (also available for the crude extracts), confirmed by lower EC_50_ values (see Table 3). Our results are in good concordance with other authors’ research, according to whom hydroalcoholic mixtures (ethanol 50–70%) increase polyphenols solubility compared to absolute alcohol [64,65], and thus contribute to superior antioxidant activity. As shown in Table 1 (HPLC analysis), some phenol carboxylic acids (mainly rosmarinic acid and chlorogenic acid) were only identified in RS1 and RS3 solutions. More than that, it can be observed that the size of the nanoparticles (for the differences recorded in our study) does not strongly influence the final antioxidant potential of the nanoparticles/extract solutions: for the same type of solvent, for sample RS1N, were recorded smaller EC_50_ values, compared with RS3N, while RS2N shows a higher EC_50_ value for the ABTS assay and smaller values for the other two assays.

Regarding the results obtained for the crude extracts, it can be observed that, for the ABTS assay, the extraction method was a key factor that influenced the antioxidant activity. The antioxidant activity was higher for RS1 compared to RS3, while RS2 had a lower antioxidant activity compared to RS4. These results are partly correlated with our spectrophotometric determinations, since RS1 had a higher phenolic content compared to RS3 (see Table 1). However, RS4 has a better antioxidant activity, although is phenolic content is much lower compared to RS2. These results could be the consequence of other compounds (amino acids, protein) interaction with Folin–Ciocalteu reagent [66]. Moreover, for HPLC analysis, independent of the solvent used, microwave-assisted extraction technique led to a higher content of phenolic compounds (chlorogenic acid, ferulic acid, rutin, rosmarinic acid, and quercetin) (see Table 1), being a valuable alternative extraction method, due to shorter extraction time, lower solvent consumption, and higher polyphenols yield [67,68]. The antioxidant activity determined by ABTS assay was higher compared to DPPH and ferric reducing power methods. These differences might be the consequence of different mechanisms of action, since DPPH and ferric reducing power are mainly electron transfer methods, while ABTS assay has a mixed mechanism (both electron and hydrogen atom transfer). DPPH and ferric reducing power are mainly used for evaluation of hydrophilic compounds antioxidant capacity, while ABTS can be used for both hydrophilic and lipophilic (triterpenes, flavones, aglycones, etc.) ones [69]. As stated by the scientific literature [26,70], polyphenols and other compounds (such as minerals, glucosinolates, vitamins) are responsible for the overall antioxidant characteristic.

The phytosynthesis of the silver nanoparticles increased the antioxidant potential of the extracts, compared to the “*parental solutions*”, for all the assays and samples studied, which is in concordance with other published studies [71,72], the best results being obtained for the silver nanoparticles phytosynthesized using the hydroalcoholic extract obtained by classical extraction (sample RS1N). The results obtained by our group in the antioxidant assays are relatively hard to compare with literature data, considering the low number of papers presenting phytosynthesis of silver nanoparticles using *R. sativus* extracts. As such, for comparison purposes were considered studies regarding the application of other plants from the *Brassicaceae* family. Ansar et al. [73] obtained an inhibition of the DPPH radical from around 50% to 79%, for the tested concentration range 50–200 μg/mL, with an EC_50_ of 50.37 μg/mL for AgNPs phytosynthesized using *Brassica oleracea* L. leaves aqueous extracts in a different extract/silver salt ratio (1/5). However, the authors did not study the antioxidant potential of the crude extract, so no consideration regarding the AgNPs contribution to the final antioxidant activity can be made. The results of the antioxidant assays are although comparable with the antioxidant properties of AgNPs phytosynthesized using other plant extracts: for example, *Prosopis farcta* mediated NPs showed IC_50_ in the DPPH assay of 0.7 mg/mL (compared with the extract—1.64 mg/mL), while in the FRAP assay, the IC_50_ was not reached in the concentration range 0.2–1 mg/mL [74], while the NPs phytosynthesized using dittany, sage, sea buckthorn, and, respectively, calendula extracts showed IC_50_ between 0.61 and 2.08 mg/mL in the DPPH assay. [75].

The results of the antioxidant assays should, however, be considered as promising towards potential application, and not as a definitive statement of their antioxidant potential. The chemical antioxidant assays are to be used as screening tools, and the obtained results should be confirmed by more specific assays, such as in vivo or cell-based models [76], subject of future works.

### 3.4. In Vitro Antimicrobial Activity

**Figure 4:**
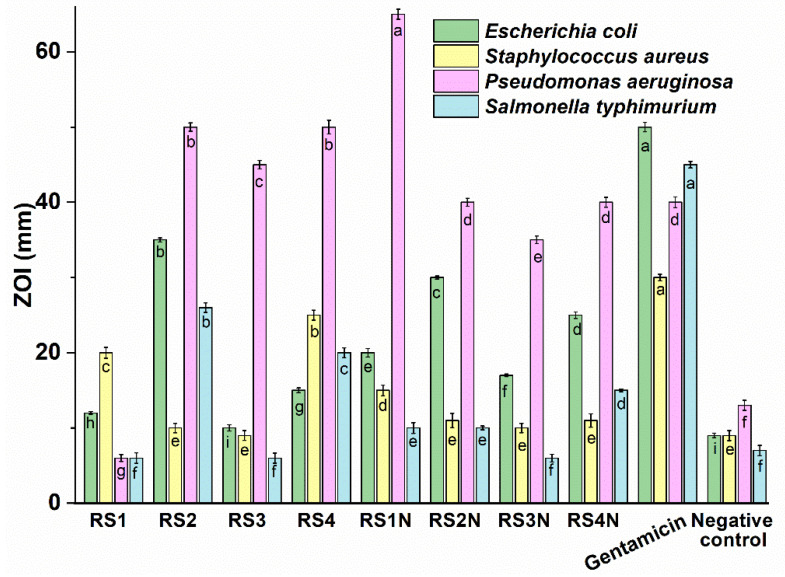
Antibacterial activity of tested extracts against four bacterial strains. For each strain, values without a common letter differ (*p* < 0.05) as analyzed by one-way ANOVA and the Tukey test.

Antibacterial activity (see Figure 4) was tested on three Gram-negative bacteria, *E. coli*, *P. aeruginosa*, and *S. typhimurium* and one Gram-positive bacterium *S. aureus*. Positive controls for the antimicrobial assays are detailed in the Supplementary Material.

The diameter of the inhibition zone for the phytosynthesized nanoparticles varied from 7.38 ± 0.21 mm (RS3N against *S. typhimurium*) to 65.67 ± 0.91 mm (RS1N against *P. aeruginosa*) for the tested samples, compared with the values obtained for the positive control gentamicin (30.33 ± 0.44 mm against *S. aureus* to 50.10 ± 0.62 mm against *E. coli*) (see Figure 4). The antibacterial activity of the tested samples found to be the highest against *P. aeruginosa* (extremely sensitive to RS1N, and with comparable values to the positive control for RS2N and RS4N), while lowest activity was found against *S. typhimurium* (not sensitive to RS3N). The antimicrobial effect of the nanoparticles is in close connection with the antimicrobial potential of the extracts used for phytosynthesis. Antimicrobial effect of phenolics products can involve various modes of action such as enzyme inhibition by the oxidized products, maybe through reaction with sulfhydryl groups or through more nonspecific interactions with the proteins [77]. Phenols can also inhibit the synthesis of nucleic acids of both Gram-positive and Gram-negative bacteria in accordance with Cushnie and Lamb [78]. *S. aureus* showed lower sensitivity to the extract as compared to the most other Gram-negative bacteria. The obtained results are in accordance with the findings of other authors [79]. In our case, most of the nanoparticles revealed an increase of the antimicrobial potential, compared with the corresponding extracts. A notable exception is the case of *P. aeruginosa,* where extracts RS2–RS4 possess higher antimicrobial potential, compared with the phytosynthesized NPs. However, sample RS1N presents a significant increase, compared with RS1, showing a higher antimicrobial potential, even when compared with the positive control. For *E. coli*, only RS2 sample have a superior antimicrobial effect compared with the NPs (RS2N), while all the other phytosynthesized nanoparticles have significant superior effects, compared with the corresponding extracts. For *S. aureus,* extracts RS1 and RS4 have superior effects, compared with the NPs, while for the other two sets, phytosynthesis does not statistically influence the antimicrobial potential, while for *S. typhimurium*, RS1N showed superior effect compared with RS1, while RS2 and RS4 presents superior effects, compared with the corresponding NPs. Phytosynthesis of silver nanoparticles does not influence the antimicrobial potential of sample RS3N.

**Figure 5:**
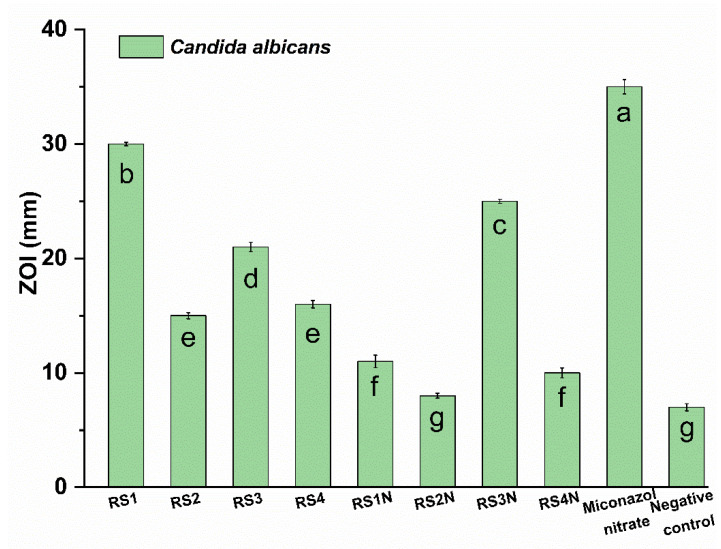
Anti-*Candida* effect of the tested samples. Values without a common letter differ (*p* < 0.05) as analyzed by one-way ANOVA and the Tukey test.

Figure 5 presents the anti-Candida properties of the evaluated materials.

**Figure 6:**
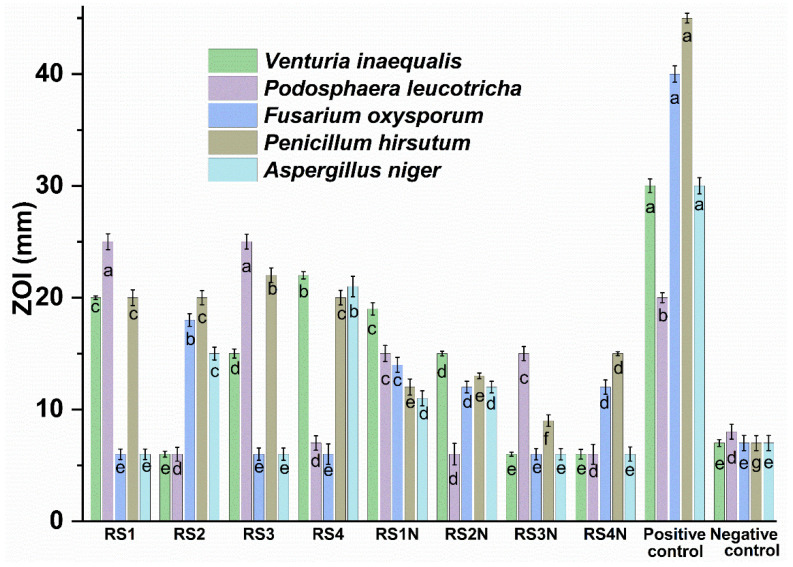
Antifungal activity of tested extracts against horticultural relevant fungi. For each strain, values without a common letter differ (*p* < 0.05) as analyzed by one-way ANOVA and the Tukey test.

The phenolic compounds have been reported in the literature as potent anti-Candida agents, their mechanism of action including inactivation of enzyme production [80]. Our results are partly correlated with the HPLC determinations. *C. albicans* is extremely sensitive at for samples RS3, RS3N, and RS1 and not sensitive for the RS2N sample. Results obtained for the antimicrobial susceptibility assay of tested samples (determined on 3 different days) showed that the extracts were only active in the undiluted form, while for the NP formulation MIC value was 53.93 ± 0.23 μg/mL (expressed as AgNP concentration) against all tested microbial strains (Data presented in Supplementary Material, Table S2, results presented as the median of 3 experiments). Figure 6 and Figure S3 (see Supplementary Material) show that the most effective nanoparticles against *V. inaequalis* and *P. leucotricha* were RS1N (against both lines), RS2N (against are *V. inaequalis*), respectively RS3N (against *P. leucotricha*). Among the crude extracts, the most effective were RS1 and RS3 (superior effect against *P. leucotricha* compared with the positive control), respectively RS4 (against *V. inaequalis*). At the same time, it was observed that pathogenic food fungi are sensitive at RS1N and RS2N, while RS3N and RS4N were effective only against *P. hirsutum*, respectively against *F. oxysporum* and *P. hirsutum*. Compared with the corresponding extracts, only sample RS1N shows enhanced activity against all pathogenic fungi.

The results obtained in the present study are comparable with the literature data: for example, the inhibition zones determined by Loo et al. [81] against *E. coli* and *S. typhimurium* for AgNPs phytosynthesized using pu-erh tea leaves were 15, respectively 20 mm, with MIC values of 7.8, respectively 3.9 μg/mL, while the MIC values of AgNPs phytosynthesized using *Brassica oleracea* recorded against *S. aureus*, *E. coli*, and *P. aeruginosa* were 25, 25, respectively 12.5 μg/mL [73]. The enhanced antimicrobial properties usually presented by the phytosynthesized nanoparticles can led to their application in important biomedical applications, such as wound dressings [82]. Another important aspect regarding the application of nanoparticles in general is represented by their potential toxicity. As the potential uses of nanoparticles extends, so does the concerns related to their safety for the environment and living creatures. In this respect, another major advantage of the phytosynthesized nanoparticles is represented by the lower toxicity exhibited, in comparison with nanoparticles obtained by other routes, with comparable size and morphology, as previously presented by our group [40,83].

According to all the results obtained (summarized in Table S3, Supplementary Material), it was observed that RS1/RS1N are the most promising formulations from all the tested samples as anti- *V. inaequalis* and *P. leucotricha* treatment. Withal, the best antibacterial couples were RS2/RS2N and RS4/RS4N; RS2/RS2N also presented the best antifungal solution, while for yeast inhibition, the best results were obtained from RS3/RS3N and RS1. Considering the results obtained for the in vitro assay regarding the isolates, as well as economic aspects (regarding both the extraction method and the solvent used), the NPs phytosynthesized with the hydroalcoholic extract and its corresponding extract (RS1N/RS1) were selected for further in vivo studies.

### 3.5. In Vivo Antifungal Activity

One of the main drawbacks related to the industrial application of AgNPs is related to the possibilities to achieve the control of the extraction procedure at the industrial scale. The recipe proposed for in vivo evaluation is not only capable to ensure the success of the extraction (not involving the use of complex equipment), but also proposes the use of economically viable solvents (such as the hydroalcoholic mixture).

**Figure 7:**
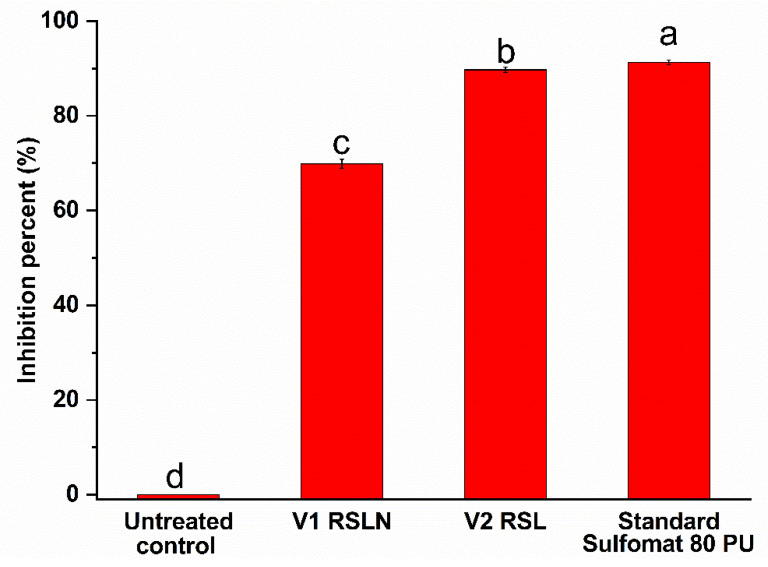
Graphical representation of the effect of tested materials in control of powdery mildew—*Podosphaera leucotricha*. Values without a common letter differ (*p* < 0.05) as analyzed by one-way ANOVA and the Tukey test.

**Figure 8:**
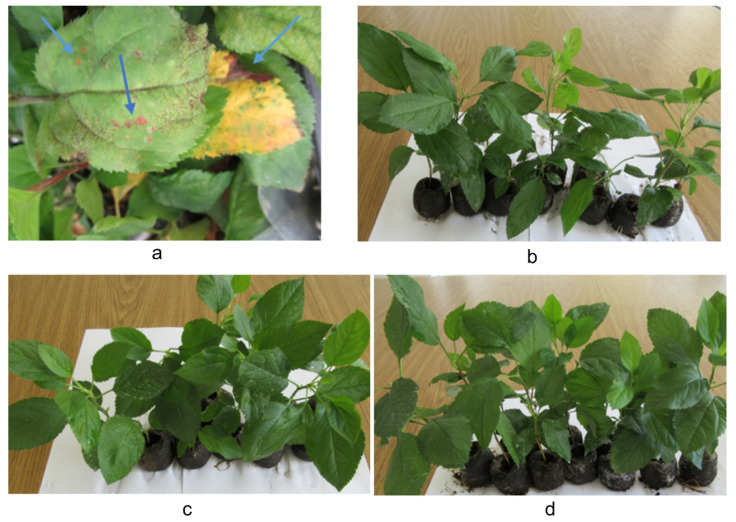
Effect of the tested materials for the control of apple scab—*Venturia inaequalis*: (**a**)—untreated (negative) control; (**b**)—RS1N; (**c**)—RS1; (**d**)—standard Captadin 80 WDG (positive control).

**Figure 9:**
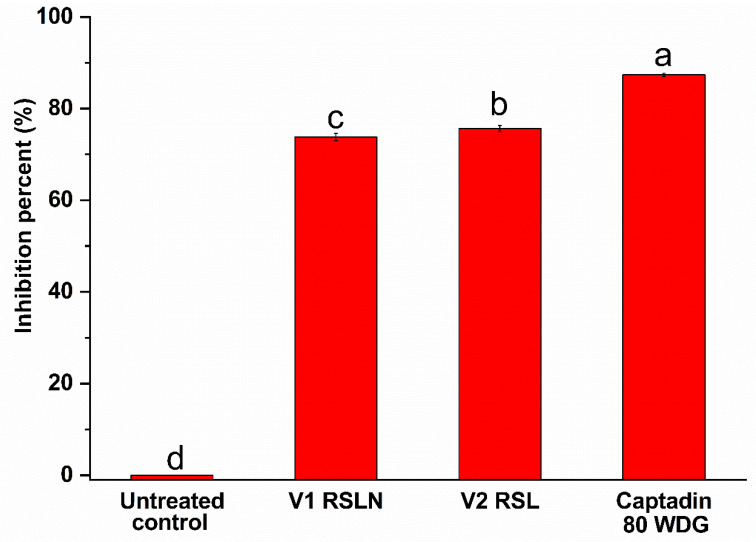
Effect of vegetal extracts in control of apple scab—*Venturia inaequalis*. Values without a common letter differ (*p* < 0.05) as analyzed by one-way ANOVA and the Tukey test.

Results of the in vivo evaluation of the RS1 and RS1N materials are presented in Table 4 and Table 5 (*P. leucotricha* experiments results graphically represented in Figure 7 and Figure S4, Supplementary material, Figure S5—representative images for *P. leucotricha*, Figure 8—representative images for *V. inaequalis*, Figure 9 and Figure S6—Supplementary Material—graphical representation of *V. inaequalis* experiments results).

Figure 7 shows that, in the variant V2 (treatment with RS1 extract), the inhibition percent of the powdery mildew—*P. leucotricha* was 89.72%, while the value obtained in the variant treated with chemical standard product Sulfomat 80 PU was 91.29%. Additionally, interesting results were obtained in the variant V1 (treatment with RS1N), where the inhibition percentage was 69.86%.

Evaluating the results presented in Table 4 and Figure S4, it can be observed that the treatments with vegetal extract RS1 were very effective in control of powdery mildew—*P. leucotricha.* The damages degree was limited at 0.59%, which was like the effectiveness of chemical standard Sulfomat 80 PU with damages degrees limited at 0.50. By comparation, in the variant V1—RS1N, the powdery mildew damage degree was 1.73%. In the untreated control variant, the powdery mildew damage degree was 5.74%.

Figure 8 presents the visual appearance of the leaves used for evaluating the effect of the tested materials for the control of apple scab (the effect being graphically presented in Figure 9). Figure 9 shows that in the variant V2 (treated with RS1 extract), the inhibition percent of the apple scab—*V. inaequalis* was 75.65%, similar to the value obtained in the V1 variant (treated with RS1N), for which the inhibition percentage was 73.76%. The inhibition value for the variant treated with standard chemical product was 87.39%.

Evaluation of the results presented in Table 5 and Figure S6 reveals that the treatments with vegetal extract RS1 was effective in control of apple scab—*V. inaequalis* the damages degree was limited at 0.56%, which was pretty close to the effectiveness of RS1N extract with damages degrees limited at 0.60. By comparison to the variant treated with standard chemical product Captadin 80 WDG, the damages degree was 1.73%. In the untreated control variant (negative control), the apple scab damage degree was 2.30%.

## 4. Conclusions

A horticultural circle was created to save other horticultural crops with the help of residues from horticulture, with the development of new “green” materials. From *Raphanus sativus* L. waste, alcoholic and hydroalcoholic extracts were obtained through the two previous established methods. These methods were successfully used for phytosynthesis of silver nanoparticles. The obtained nanoarchitectures were investigated from the antioxidant and antimicrobial point of view to demonstrate their potential application for controlling apple crops pathogens. The antioxidant assays revealed that phytosynthesized silver nanoparticles showed a higher antioxidant activity compared to the crude extracts; also, the nanoparticles phytosynthesized using hydroalcoholic extracts constantly presented superior results, compared with the ones obtained using ethanol as solvent. The influence of the silver nanoparticles on the final antioxidant potential was minimal, most probably due to the relatively small differences in terms of particle size.

The antibacterial activity of the phytosynthesized nanoparticles was found highest against *P. aeruginosa* (extremely sensitive to all phytosynthesized nanoparticles, especially RS1N, superior effect compared with the positive control), while the lowest activity was found against *S. typhimurium* (not sensitive to RS3N; an increase of the antimicrobial efficiency, compared with the corresponding extract being observed for sample RS1N). *C. albicans* is extremely sensitive to RS3N and not sensitive to the RS2N sample, while *V. inaequalis* and *P. leucotricha* are extremely sensitive in vitro to RS1N; at the same time, it is observed that pathogenic food fungi are sensitive at RS1N and RS2N. Considering the obtained results for the in vitro assay, as well as economic aspects (regarding both the extraction method and the solvent used), the NPs phytosynthesized using the extract RS1 and its corresponding crude extract (RS1N/RS1) were selected for the in vivo assay on young seedlings originated from “Idared” cvs. The results of the performed tests against *V. inaequalis* and *P. leucotricha* allow us to propose a nanotechnological approach for the development of potential horticultural formulations, in order to surpass one of the main drawbacks related to the application of phytosynthesized nanoparticles, respectively their production at an industrial scale, for large-scale application; in the same time, residues from primary and secondary processing were minimized and reutilized, in order to contribute to the sustainability needs of new materials.

## Figures and Tables

**Table 1:** Total phenolic contents and HPLC identification and quantification of components of the crude extracts of the leaves of *Raphanus sativus* L. *.

Crude Extracts	Total Phenols (mg GAE/g DW)	Chlorogenic Acid (μg/mL)	Ferulic Acid (μg/mL)	Rutin (μg/mL)	Rosmarinic Acid (μg/mL)	Quercetin (μg/mL)	Chalcone (μg/mL)
RS1	12.42 ± 0.72 ^a^	41.024 ± 0.031 ^b^	53.163 ± 0.022 ^b^	105.954 ± 0.03 ^b^	0.0993 ± 0.001 ^b^	6.585 ± 0.012 ^b^	12.749 ± 0.014 ^a^
RS2	5.32 ± 0.21 ^c^	-	1.074 ± 0.003 ^d^	3.104 ± 0.013 ^d^	-	-	1.665 ± 0.009 ^d^
RS3	8.98 ± 0.37 ^b^	81.048 ± 0.053 ^a^	90.443 ± 0.041 ^a^	279.912 ± 0.04 ^a^	0.156 ± 0.001 ^a^	20.655 ± 0.015 ^a^	5.741 ± 0.011 ^b^
RS4	4.49 ± 0.18 ^c^	-	50.632 ± 0.028 ^c^	11.12 ± 0.021 ^c^	-	2.718 ± 0.011 ^c^	2.710 ± 0.009 ^c^

* Values are means ± SE, *n* = 3 per group. Means in a column without a common superscript letter (a to c) differ (*p* < 0.05) as analyzed by one-way ANOVA and the Tukey test; GAE—gallic acid equivalents; DW—dry weight.

**Table 2:** Analysis of XRD and TEM data.

Scheme.	Analysed Peak	FWHM (Full Width at Half Maximum)—XRD	Crystallite Size (nm)—XRD	Average Diameter (nm)—TEM
RS1N	200	0.784	11.51	12
RS2N	200	0.957	9.43	11
RS3N	200	0.525	17.16	16
RS4N	200	0.607	14.87	15

**Table 3:** Evaluation of antioxidant activity for analyzed extracts and silver nanoparticles *.

Extract/Nanoparticles	Method—EC_50_ (mg/mL)		
	DPPH	ABTS	Ferric Reducing Power
RS1	3.0368 ± 0.2536 ^e,f^	0.3972 ± 0.00277 ^d^	1.6028 ± 0.1250 ^c^
RS1N	1.9746 ± 0.1281 ^f^	0.3478 ± 0.02998 ^d^	1.3976 ± 0.1396 ^c^
RS2	39.065 ± 0.4315 ^a^	49.85 ± 0.1296 ^a^	10.17 ± 0.8414 ^a^
RS2N	21.790 ± 0.8768 ^d^	12.37 ± 0.7484 ^c^	8.4650 ± 0.2333 ^b^
RS3	3.7026 ± 0.1232 ^e^	0.8801 ± 0.1302 ^d^	1.9610 ± 0.0091 ^c^
RS3N	3.3596 ± 0.0738 ^e^	0.3582 ± 0.0273 ^d^	1.9565 ± 0.1851 ^c^
RS4	29.35 ± 0.3676 ^b^	23.67 ± 2.0718 ^b^	10.34 ± 0.2404 ^a^
RS4N	27.015 ± 0.8280 ^c^	11.54 ± 1.47077 ^c^	10.20 ± 0.1838 ^a^

* Values are means ± SE, *n* = 3 per treatment group. Means in a column without a common superscript letter (a–f) differ (*p* < 0.05) as analyzed by one-way ANOVA and the Tukey test.

**Table 4:** Effect of vegetal extracts in control of powdery mildew—*Podosphaera leucotricha.*

Variant/Product Name	Replication	Assessed Leaves (no)	Attacked Leaves (no)	Frequency [F%]	Intensity (I) [Scale 1–6]	Damage Degree DD [%]	Inhibition Percent [%]
Untreated control	R1_R3	46	44	95.60	6.00	5.74	0.00
V1 RS1n	R1_R3	48	22	46.76	3.70	1.73	69.86
V2 RS1	R1_R3	48	14	29.30	2.00	0.59	89.72
Standard Sulfomat 80 PU	R1_R3	50	12	23.80	1.80	0.50	91.29
	AVG_v	48.,67	16.00	33.29	2.50	0.94	83.62
Indicators	STDEV	1.1547	5.2915	11.9879	1.0440	0.6856	11.9452
	VAR	2.3727	33.0719	36.0142	41.7612	72.9403	14.2846

**Table 5:** Effect of vegetal extracts in control of apple scab—*Venturia inaequalis*.

Variant/Product Name	Replication	Assessed Leaves (no)	Attacked Leaves (no)	Frequency [F%]	Intensity (I) [Scale 1–6]	Damage Degree DD [%]	Inhibition Percent [%]
Untreated control	R1_R3	47	29	62.37	3.67	2.30	0.00
V1 RS1N	R1_R3	51	16	21.94	2.50	0.60	73.76
V2 RS1	R1_R3	47	24	37.53	1.50	0.56	75.65
Captadin 80 WDG	R1_R3	52	17	21.94	1.33	0.29	87.39
	AVG_v	50.00	19.00	27.14	1.78	0.48	78.93
Indicators	STDEV	2.6458	4.3589	9.0007	0.6322	0.1698	7.3844
	VAR	5.2915	22.9416	33.1642	35.5816	35.0467	9.3552

## Data Availability

All data will be made available upon request.

## Supplementary Materials

The following are available online at https://www.mdpi.com/article/10.3390/ma14081845/s1. The Supplementary Material provided contain the protocols followed for the antioxidant assays, the disease severity rating scale used for the in vivo assay, as well as results obtained for the analytical characterization, in vitro and in vivo assays.
