# Supplementary material for: In Vitro and In Vivo Evaluation of Silver Nanoparticles Phytosynthesized Using Raphanus sativus L. Waste Extracts
Source: Materials (Basel). 2021 Apr 8;14(8):1845. doi: 10.3390/ma14081845 (PMC8068147; doi:10.3390/ma14081845)
Supplement: Supplementary file 1 [file materials-14-01845-s001.pdf]

materials

Supplementary

In Vitro and In Vivo Evaluation of Silver Nanoparticles Phyto-synthesized Using *Raphanus sativus* L. Waste Extracts

Camelia Ungureanu [1,*], Irina Fierascu [2,3], Radu Claudiu Fierascu [2,4,*], Teodora Costea [5], Sorin Marius Avramescu [6], Mirela Florina Călinescu [7], Raluca Somoghi [2] and Cristian Pirvu [1]

Citation: Ungureanu, C.; Fierascu, I.; Fierascu, R.C.; Costea, T.; Avramescu, S.M.; Călinescu, M.F.; Somoghi, R.; Pirvu, C. In Vitro and In Vivo Evaluation of Silver Nanoparticles Phytosynthesized Using *Raphanus sativus* L. Waste Extracts. *Materials* **2021**, *14*, 1845. https://doi.org/10.3390/ma14081845

Academic Editor: Rosaria Anna PICCA

Received: 26 March 2021
Accepted: 06 April 2021
Published: 08 April 2021

Publisher's Note: MDPI stays neutral with regard to jurisdictional claims in published maps and institutional affiliations.

Copyright: © 2021 by the authors. Submitted for possible open access publication under the terms and conditions of the Creative Commons Attribution (CC BY) license (http://creativecommons.org/licenses/by/4.0/).

[1] Department of General Chemistry, University "Politehnica" of Bucharest, 011061 Bucharest, Romania; cristian.pirvu@upb.ro
[2] National Institute for Research & Development in Chemistry and Petrochemistry—ICECHIM, 060021 Bucharest, Romania; irina.fierascu@icechim.ro (I.F.); fierascu.radu@icechim.ro (R.C.F.); raluca.somoghi@icechim.ro (R.S.)
[3] Faculty of Horticulture, University of Agronomic Sciences and Veterinary Medicine of Bucharest, 011464 Bucharest, Romania
[4] Department of Science and Engineering of Oxide Materials and Nanomaterials, University "Politehnica" of Bucharest, 011061 Bucharest, Romania
[5] Phytotherapy Department, Faculty of Pharmacy, Pharmacognosy, Phytochemistry, University of Medicine and Pharmacy "Carol Davila", 020956 Bucharest, Romania; teodora.costea@umfcd.ro
[6] Research Center for Environmental Protection and Waste Management, University of Bucharest, 050107 Bucharest, Romania; sorin_avramescu@yahoo.com
[7] Research Institute for Fruit Growing Pitesti—Mărăcineni, 110006 Pitesti-Mărăcineni, Romania; elacalinescu@yahoo.com
* Correspondence: camelia.ungureanu@upb.ro (C.U.); fierascu.radu@icechim.ro (R.C.F.)

1. Experimental procedures

1.1. Evaluation of chemical composition of radish leaf extracts

Evaluation of total phenolic content of the extracts

For the quantification of the total phenolic content, 150 µL of 10% diluted Folin-Ciocalteu reagent and 120 µL of 0.7 M Na_2CO_3 were added to 30 µL of the extracts dissolved in their solvents (240 µg/mL) and the mixtures were incubated for 30 min with shaking at room temperature. Absorbance was spectrophotometrically measured at 765 nm and the results expressed as milligrams of gallic acid equivalents (GAE) per gram of dry weight using Equation S1:

$$C_{tp} = c \times \frac{V}{m} \quad \text{(S1)}$$

where: C_{tp}—total phenolic content (mg/g) in GAE (gallic acid) equivalent, C—Concentration of gallic acid obtained from calibration curve in mg/mL, V- volume of extract in mL, m—mass of extract in grams.

The calibration curves were constructed using analytic standards gallic acid (Sigma-Aldrich, Germany). The experiments were carried out in triplicate.

1.2. Evaluation of biological properties

1.2.1. DPPH assay

DPPH is a stable free radical, at ambient temperature, presenting strong absorbance at 517 nm; in the presence of an antioxidant, it is reduced, the solution becomes yellow to colorless and the absorbance decreases. For the assay, 0.5 mL of different dilutions from analysed extracts or silver nanoparticles (concentration range 0.8–7.2 mg/mL) were mixed

with 3 mL of 0.1 mM DPPH ethanolic solution. The mixture was kept in the dark at room temperature and the absorbance of the DPPH solution was measured at λ = 517 nm before (A_{start}) and 30 min. after adding the test solutions (A_{end}). Ethanol was used as blank. The ability to scavenge the DPPH free radical was calculated using Equation S2:

$$\text{DPPH radical scavenging activity (\%)} = \frac{A_{start} - A_{end}}{A_{start}} \times 100 \tag{S2}$$

The extracts concentration that inhibited 50% of the DPPH free radical (EC_{50}, mg/mL) was determined graphically from the linear regression curve plotted between percent (%) of inhibition and solutions concentration (mg/mL). All measurements were performed in triplicate.

1.2.2. ABTS•+ radical cation scavenging assay

The ABTS radical cation was generated by incubation of ABTS diammonium salt (7 mM) with potassium persulfate (2.45 mM) in the dark, at room temperature for 16 h. The absorbance of the ABTS radical solution was equilibrated to a value of 0.700 ± 0.02 at λ = 734 nm after dilution with ethanol. 0.3 mL of several dilutions of analyzed extracts or silver nanoparticles (concentration range 0.004–0.6 mg/mL) were mixed with 3 mL reagent and the absorbance of the ABTS•+ cation radical was measured at λ = 734 nm, before ($A_{control}$) and 6 min. after adding the test solutions (A_{sample}). The scavenging activity was calculated using Equation S3:

$$\text{ABTS radical scavenging activity (\%)} = \frac{A_{control} - A_{sample}}{A_{control}} \times 100 \tag{S3}$$

The extracts concentration that inhibited 50% of the ABTS•+ free radical (EC_{50}, mg/mL) was determined graphically from the linear regression curve plotted between percent (%) of inhibition and solutions concentration (mg/mL). All measurements were performed in triplicate.

1.2.3. Ferric reducing power assay

For the assay, 2.5 mL of several dilutions of analyzed extracts or silver nanoparticles (concentration range 0.8–7.2 mg/mL) were mixed with 2.5 mL of 0.2 M phosphate buffer (pH 6.6) and 2.5 mL of 1% potassium ferricyanide. Samples were kept at 50 °C in a water bath (Raypa, Spain) for 20 min. After, 2.5 mL of 10% trichloroacetic acid was added and the mixture was centrifuged at 2500 rpm for 5 min. (Universal 16 centrifuge). The upper layer (2.5 mL) was mixed with 2.5 mL water and 0.5 mL of a 0.1% ferric chloride solution. The absorbance was measured at λ = 700 nm, after 10 min., against a blank that contained all reagents except the tested solutions. A higher absorbance indicates a stronger reducing power.

The solutions concentration providing 0.5 of absorbance (EC_{50}, mg/mL) was determined graphically from the linear regression curve plotted between absorbance and solutions concentrations (mg/mL). All measurements were performed in triplicate.

1.3. In vivo antifungal activity

Young seedlings, originated from "Idared" cvs. were inoculated in greenhouse with a suspension of 4.5 × 10^5 conidia/mL of *V. inaequalis* or *P. leucotricha* applied using a manual atomizer under controlled conditions (temperature 18–20 °C and humidity 80–100 %). The conidial suspension was provided from the naturally infected leaves of apples of three varieties: "Idared", "Starkrimson" and "Golden Delicious" cvs. from demonstrative apple plots of Research Institute for Fruit Growing Pitesti, Romania. The leaves were collected in 2018, from August to September, dried and kept in storage. The leaves were hydrated in distilled water and the inoculum of *V. inaequalis* or *P. leucotricha* was brought to 4.5 × 10^5 conidia/mL. After primary infection, the treatments with the developed materials were applied. The experimental variants were tested in triplicate.

Visual assessment regarding powdery mildew leaf area infection was carried out with the aid of the diseases rating scale, as presented in Table S1.

Table S1. Powdery mildew—*Podosphaera leucotricha* disease severity rating scale for leaves.

Powdery mildew disease rating	Percent of leaf area infected
0	No infection
1	≤1% infection
2	2–5% infection
3	6–20% infection
4	21–40% infection
5	>40% infection
6	100% infection

Damage degree DD% was calculated according to Equation S4:

$$DD\ (\%) = \frac{F \times I}{100} \quad (S4)$$

where: DD (%) = damage degree; F = attack frequency, %; I = attack intensity, estimated with notes between 1 and 6.

The antifungal efficacy of the vegetal extracts on the diseases was evaluated after 14 days, as a percentage of inhibition calculated using Abbott Equation (S5):

$$\text{Percent inhibition}\ (\%) = \frac{A - B}{A} \times 100 \quad (S5)$$

where: A and B represent the diseases area on the untreated and, respectively, treated plants.

2. Results

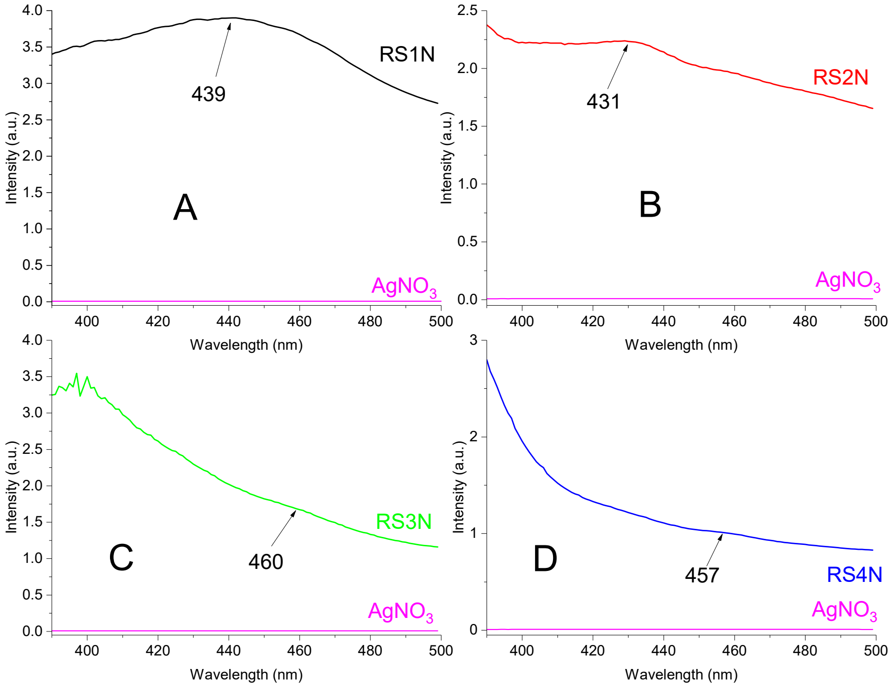

Figure S1. UV-Vis spectra of the phytosynthesized NPs, compared with the silver nitrate solution. (**A**): RS1N; (**B**): RS2N; (**C**): RS3N; (**D**): RS4N.

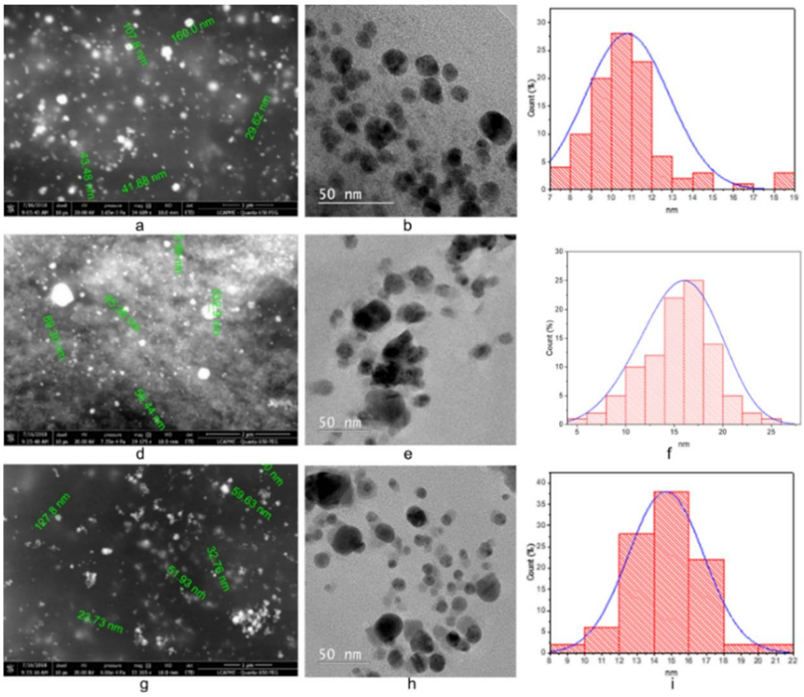

Figure S2. SEM, TEM and size distribution of the phytosynthesized nanoparticles: a–c—sample R2N, d–f—sample R3N, g–i—sample R4N.

Table S2. Results of Minimum Inhibitory Concentrations (MICs) concerning antimicrobial susceptibility of the microorganisms to tested extracts and nanoparticle formulations.

**Microorganism**	**MIC (μg/mL)**							
	**RS1**	**RS1N**	**RS2**	**RS2N**	**RS3**	**RS3N**	**RS4**	**RS4N**
Escherichia coli ATCC 8738	Undiluted	53.93 ± 0.23	Undiluted	53.93 ± 0.23	Undiluted	53.93 ± 0.23	Undiluted	53.93 ± 0.23
Staphylococcus aureus ATTC 25923	Undiluted	53.93 ± 0.23	Undiluted	53.93 ± 0.23	Undiluted	53.93 ± 0.23	Undiluted	53.93 ± 0.23
Pseudomonas aeruginosa ATCC 9027	Undiluted	53.93 ± 0.23	Undiluted	53.93 ± 0.23	Undiluted	53.93 ± 0.23	Undiluted	53.93 ± 0.23
Salmonella typhimurium ATCC 14028	Undiluted	53.93 ± 0.23	Undiluted	53.93 ± 0.23	Undiluted	53.93 ± 0.23	Undiluted	53.93 ± 0.23
Venturia inaequalis (isolates)	Undiluted	53.93 ± 0.23	Undiluted	53.93 ± 0.23	Undiluted	53.93 ± 0.23	Undiluted	53.93 ± 0.23
Podosphaera leucotricha (isolates)	Undiluted	53.93 ± 0.23	Undiluted	53.93 ± 0.23	Undiluted	53.93 ± 0.23	Undiluted	53.93 ± 0.23
Fusarium oxysporum ATCC 48112	Undiluted	53.93 ± 0.23	Undiluted	53.93 ± 0.23	Undiluted	53.93 ± 0.23	Undiluted	53.93 ± 0.23
Penicillum hirsutum ATCC 52323	Undiluted	53.93 ± 0.23	Undiluted	53.93 ± 0.23	Undiluted	53.93 ± 0.23	Undiluted	53.93 ± 0.23
Aspergillus niger ATCC 15475	Undiluted	53.93 ± 0.23	Undiluted	53.93 ± 0.23	Undiluted	53.93 ± 0.23	Undiluted	53.93 ± 0.23

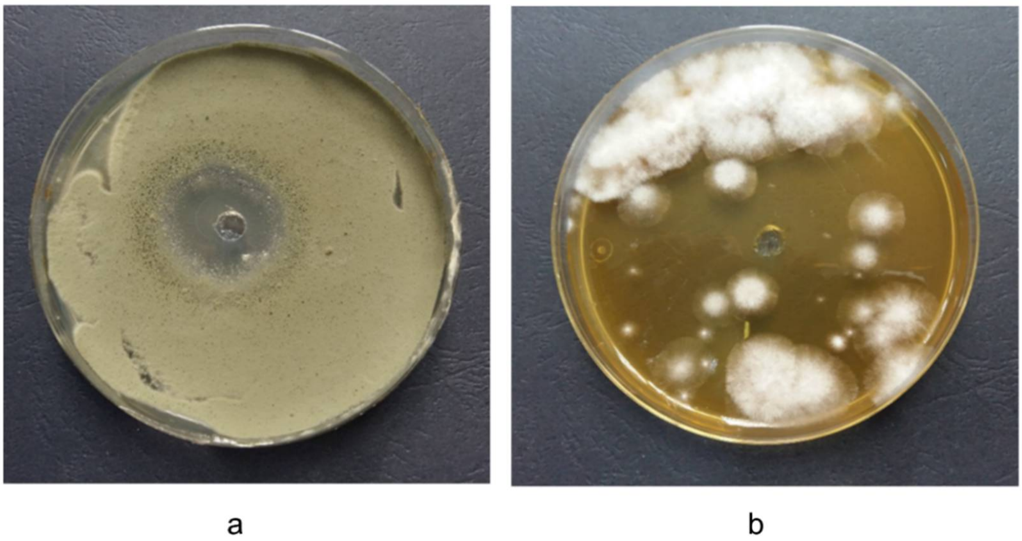

Figure S3. Representative Petri dishes for antifungal activity of RS1 on **a**) *Venturia inaequalis* and **b**) *Podosphaera leucotricha*.

Table S3. Percent inhibition for all tested samples.

Microorganism	Percent inhibition, %							
	RS1	RS2	RS3	RS4	RS1N	RS2N	RS3N	RS4N
Escherichia coli ATCC 8738	25	74	10	55	55	70	47	64
Staphylococcus aureus ATTC 25923	55	10	0	40	40	18	10	18
Pseudomonas aeruginosa ATCC 9027	0	68	71	80	80	68	63	68
Salmonella typhimurium ATCC 14028	0	73	0	30	30	30	0	53
Venturia inaequalis (isolates)	65	0	54	64	64	53	0	0
Podosphaera leucotricha (isolates)	68	0	68	47	47	0	47	0
Fusarium oxysporum ATCC 48112	0	61	0	50	50	42	0	42
Penicillum hirsutum ATCC 52323	65	65	68	42	42	46	22	53
Aspergillus niger ATCC 15475	0	53	0	36	36	42	0	0

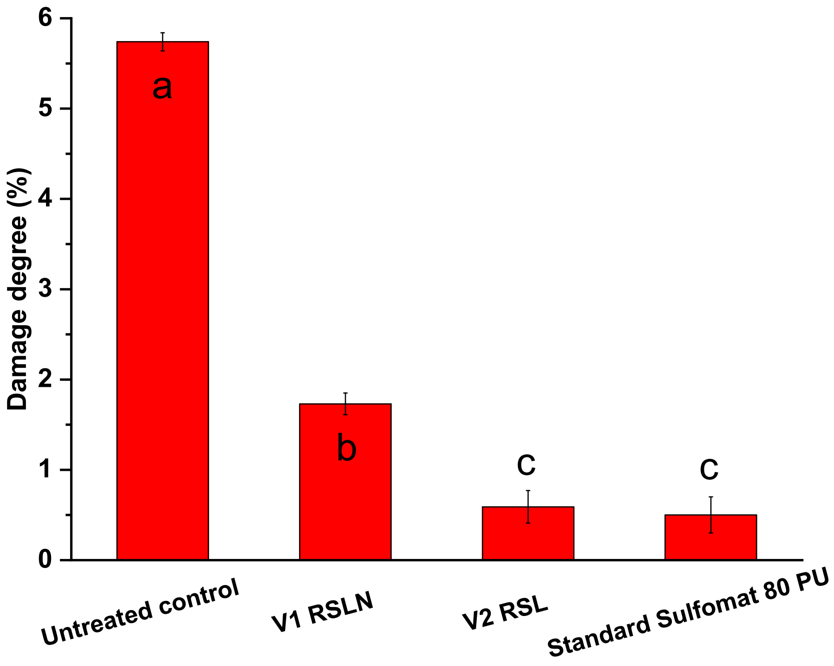

Figure S4. Effect of vegetal extracts in powdery mildew control—*Podosphaera leucotricha.* Values without a common letter differ ($p < 0.05$) as analyzed by one-way ANOVA and the TUKEY test.

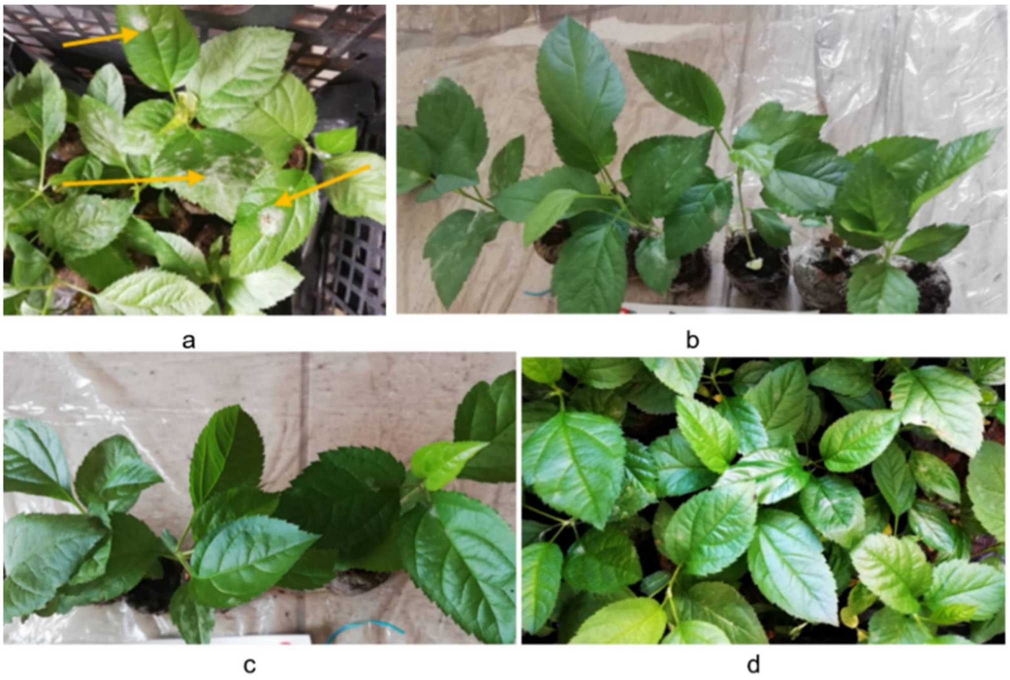

Figure S5. Effect of the tested materials for the powdery mildew control—*Podosphaera leucotricha*: —untreated (negative) control; **b**—RS1N; **c**—RS1; **d**—Standard Sulfomat 80 PU (positive control).

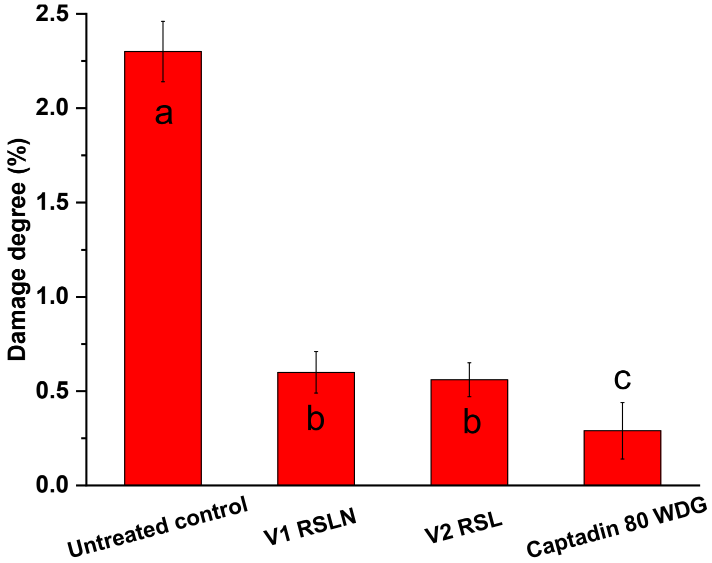

Figure S6. Effect of vegetal extracts in control apple scab—*Venturia inaequalis.* Values without a common letter differ ($p < 0.05$) as analyzed by one-way ANOVA and the TUKEY test.
